# Effects of Percutaneous Nephrolithotomy on Renal Morphology and Arterial Vascular Resistance

**DOI:** 10.7759/cureus.97673

**Published:** 2025-11-24

**Authors:** Sachin Raj, Thota Siddhartha, Joseph Philipraj, Suvit Jumde

**Affiliations:** 1 Urology, Mahatma Gandhi Medical College and Research Institute, Pondicherry, IND

**Keywords:** color doppler ultrasonography, percutaneous nephrolithotomy, renal morphology, renal resistive index, urolithiasis

## Abstract

Introduction

Urolithiasis is the most common disorders seen in urology clinics around the globe, leading surgeons to adopt percutaneous nephrolithotomy (PCNL) as the standard approach for treating large stones. However, data about how this procedure alters kidney structure and blood flow, particularly arterial resistance, remain surprisingly scarce. The present study therefore set out to measure the effects of PCNL on renal anatomy and vascular resistance by means of grey scale and color Doppler ultrasound.

Methodology

At Mahatma Gandhi Medical College and Research Institute, Puducherry, a cross-sectional study was conducted from October 2022 to April 2024. Forty patients who had PCNL for large stones agreed to participate in the study with 12-18-month postoperative assessment. Trained radiologists recorded resistive index (RI), parenchymal thickness, and echogenicity with color Doppler imaging. Results were analysed with standard statistical tests, considering p-values smaller than 0.05 as significant.

Results

The sample contained 40 patients whose average age was 43.8 ± 12.7 years; male subjects made up 72.5% (n = 29) and female subjects 27.5% (n = 11) of the cohort. The renal RI measured in the treated kidney averaged 0.624 ± 0.040, while the corresponding value for the contralateral kidney was 0.611 ± 0.037. Normal echogenicity was noted in 97.5% (n = 39) of patients, with only 2.5% (n = 1) displaying hyperechogenic areas. Serum creatinine levels averaged 1.14 ± 0.42 mg/dL, underscoring overall preserved renal function. No statistically significant differences emerged between the operated and contralateral kidneys in vascular resistance.

Conclusion

Taken together, these data suggest that PCNL exerts trivial effects on renal structure and arterial resistance during a 12-18-month follow-up period. The procedure can therefore be regarded as safe, having maintained both renal function and morphology throughout a follow-up period of 12 to 18 months.

## Introduction

Urolithiasis is the most widespread urological disorder, afflicting millions of patients worldwide each year. Clinicians regard it as a troublesome disease because stones tend to recur, and untreated cases can lead to severe complications such as hydronephrosis, infection, and permanent renal damage [1,2]. Patients typically present with intense flank pain, hematuria, and obstructive symptoms; recurrent episodes raise the risk of progressive renal impairment, underscoring the need for durable treatment strategies that preserve renal function [3].

Percutaneous nephrolithotomy (PCNL) is the preferred modality as suggested by most guidelines for managing large renal stones greater than 2 cm [4,5]. The approach is minimally invasive and achieves effective stone clearance as demonstrated by multiple studies [6]. While complications are minimal with modern PCNL techniques, questions remain about its longer-term impact on kidney structure and function [7,8]. Traditionally, post-PCNL renal performance has been judged by serum creatinine concentration and estimated glomerular filtration rate. These laboratory markers, however, might overlook subtle alterations in kidney morphology or vascular perfusion that could manifest clinically over time [9,10]. In this context, grey scale and color Doppler ultrasonography offers a safe, bedside imaging tool that quantifies renal blood-flow resistance, thereby yielding complementary information about the organ's functional well-being [11,12].

The Renal Resistive Index (RI) serves as an important indicator of renal arterial perfusion and vascular resistance. Tublin et al. established that RI serves as a reliable predictor of renal vascular health, with normal RI values typically ranging from 0.58 to 0.70 [13]. Elevated values suggest increased vascular resistance and potential impairment in renal perfusion [13,14]. Kidneys that exhibit scarring often demonstrate higher RI values, indicating compromised vascular supply and potential for progressive renal impairment [15]. In addition, renal parenchymal echogenicity and thickness provide valuable information about renal morphology, with hyperechogenic appearance often indicating chronic parenchymal disease [16]. Despite the presumed minimal impact of PCNL on renal function due to its minimally invasive nature, there remains a paucity of data concerning its effects on renal morphology and arterial vascular resistance [17,18]. Most existing studies focus on immediate postoperative outcomes and short-term complications, with limited investigation into morphological changes during extended follow-up [19]. This knowledge gap necessitates comprehensive evaluation of PCNL's impact on renal architecture, morphology, and perfusion to ensure optimal patient outcomes and informed clinical decision-making.

This investigation was designed to address this knowledge deficit by applying grey scale and color Doppler ultrasonography in a 12-18-month follow-up of kidneys treated by PCNL. The authors measured resistive index, parenchymal thickness, and echogenicity in the operated kidney and compared these metrics with the contralateral unaffected kidney, thereby generating a paired, within-subject design that strengthens the validity of the conclusions. By correlating these sonographic markers with clinical outcomes, the study aims to clarify whether PCNL alters renal architecture during this follow-up period or maintains renal morphology and vascular resistance, a distinction critical for guiding both patient counseling and surgical technique refinement.

## Materials and methods

Study design

This cross-sectional investigation took place in the Department of Urology at Mahatma Gandhi Medical College and Research Institute, Puducherry, over an 18-month period from October 2022 to April 2024. The study protocol received prior approval from the institutional ethics committee.

Inclusion criteria

Eligible for inclusion were patients who underwent PCNL for renal calculus at the study centre between October 2022 and April 2024. Only those who attended the postoperative imaging assessment at 12 to 18 months. This cross-sectional study examined patients at a single postoperative time point rather than longitudinally.

Exclusion criteria

Patients were ruled out if they had PCNL performed on a solitary kidney, were lost to follow-up during the observation period, or any procedure was done on the contralateral kidney. Individuals who declined to participate or whose medical records or imaging data were incomplete were also excluded from the final analysis.

Sample size calculation

Sample size estimation relied on earlier reports indicating a parenchymal thickness of 11.6 ± 2.58 mm. Adopting a significance level of 0.05, an 80% power, and a target relative precision of 10% of the mean, approximately 1.16 mm, the minimum number of participants required was calculated as 40 patients by the standard formula used for cross-sectional studies.

Methodology

With prior authorisation from the institutional ethics committee, patients were recruited according to clearly defined eligibility criteria and a structured data-collection plan. Clinical assessment included stone burden characteristics, presence of hydronephrosis, symptom duration, detailed medical history, standard physical examination, and recordings of preoperative and postoperative parameters. Comorbidities were specifically assessed for diabetes mellitus, hypertension, chronic kidney disease, chronic obstructive pulmonary disease, and coronary artery disease.

Renal arterial flow was evaluated with colour Doppler ultrasonography. Resistive index measurements were obtained from interlobar and arcuate arteries in the mid-kidney region using standardized technique, with values averaged from three separate measurements per kidney, following a standardized imaging protocol that included dedicated vascular-Doppler studies. All examinations were carried out by experienced radiologists (n = 2, with eight and 12 years of ultrasound experience, respectively) to ensure consistency, with both operated and contralateral kidneys compared side by side. Routine laboratory work-up included serum creatinine, urine culture and sensitivity, and any further tests judged necessary by the clinical team. Written informed consent was secured in each persons preferred language, confirming that the study aims, procedures, and risks were fully understood.

Study parameters

The study examined renal morphological changes following PCNL procedures. Primary outcome measures included renal echogenicity assessed qualitatively as normal or hyperechoic based on ultrasonographic appearance, renal resistive index measured quantitatively using color Doppler ultrasonography for both operated and contralateral kidneys, and parenchymal thickness measured in millimeters for both kidneys to assess morphological changes. Additional study parameters included demographic characteristics, comorbidity profiles, and clinical parameters including serum creatinine levels and urine culture results. Demographic characteristics included age, sex, and body mass index. Comorbidity profiles encompassed diabetes mellitus, hypertension, chronic kidney disease, chronic obstructive pulmonary disease, and coronary artery disease.

Statistical analysis

Data were collected with structured case record forms, and later entered into Microsoft Excel (Microsoft Corp., Redmond, WA, USA) while strict measures were taken to protect patient confidentiality at every stage. Each variable type then guided the choice of statistical test. Descriptive statistics, independent t-tests for continuous variables, and chi-square tests for categorical variables were applied, with p less than 0.05 set as the cut-off for statistical significance. Qualitative variables like echogenicity, renal morphology, resistive index categories, and sex were summarized with frequency and percentage. Quantitative variables like age, resistive index values, and parenchymal thickness were expressed as mean and standard deviation.

Ethical deliberations

The study adhered to the Declaration of Helsinki and to Good Clinical Practice. Approval was secured from the Institutional Human Ethics Committee of Mahatma Gandhi Medical College Research Institute, Puducherry (registration no. MGMCRI/Res/01/2023/4/IHEC/10, dated February 16, 2023) before participant recruitment. Written informed consent was obtained from each participant, and strict measures were taken to protect anonymity. Data were securely stored on password-protected computers with encrypted storage accessible only to authorized research personnel, thereby safeguarding confidentiality throughout the study.

## Results

The study successfully recruited 40 patients who satisfied the inclusion criteria and completed the designated follow-up interval. Baseline demographic and clinical characteristics are presented in Table 1. The mean age was 43.8 ± 12.6 years, with male predominance (72.5%, n = 29).

**Table 1:** Comprehensive baseline demographic and clinical characteristics of patients undergoing percutaneous nephrolithotomy: a cohort analysis (n = 40)

Characteristic	Category	Number (n = 40)	Percentage (%)
Age (years)	<30	6	15.0
	31–45	16	40.0
	46–60	13	32.5
	>60	5	12.5
	Mean ± SD	43.77 ± 12.668	-
Gender	Male	29	72.5
	Female	11	27.5
Laterality	Left	22	55.0
	Right	18	45.0
BMI	Normal	36	90.0
	Elevated	4	10.0
Preoperative stenting	Yes	10	25.0
	No	30	75.0
Urine culture	Sterile	33	82.5
	Positive	7	17.5
Renal echogenicity	Normal	39	97.5
	Hyperechoic	1	2.5

The mean renal resistive index of the operated kidney was 0.626 ± 0.042, falling within the normal physiological range (0.58-0.70) (Table 2). The contralateral kidney demonstrated a mean resistive index of 0.613 ± 0.040, also within normal limits (Table 2). Parenchymal thickness measurements showed similar values between operated (16.08 ± 1.05 mm) and contralateral kidneys (16.07 ± 0.96 mm). Statistical comparison between operated and contralateral kidneys revealed no significant differences in resistive index values (0.626 vs. 0.613, p > 0.05) or parenchymal thickness measurements (16.08 mm vs. 16.07 mm, p > 0.05) (Table 2).

**Table 2:** Comparative analysis of renal function and morphological parameters between operated and contralateral kidneys at 12-18 months post-PCNL

Parameter	Mean ± SD	Range	Normal reference
Serum creatinine (mg/dL)	1.14 ± 0.42	0.71–3.10	0.6–1.2
Resistive Index – operated kidney	0.626 ± 0.042	0.54–0.70	0.58–0.70
Resistive Index – contralateral kidney	0.613 ± 0.040	0.52–0.70	0.58–0.70
Parenchymal thickness – operated kidney (mm)	16.08 ± 1.05	14.02–17.88	15–20
Parenchymal thickness – contralateral kidney (mm)	16.07 ± 0.96	14.20–17.66	15–20

Ultrasonographic evaluation revealed normal renal echogenicity in 97.5% (n = 39) of patients (Table 1). Only one patient (2.5%, n = 1) demonstrated hyperechogenic changes, which correlated with pre-existing chronic kidney disease, compared to the contralateral kidney which showed normal echogenicity.

Clinical parameters

The mean serum creatinine level was 1.14 ± 0.42 mg/dL (Table 2). Preoperative urine cultures were sterile in 82.5% (n = 33) of cases, while 17.5% (n = 7) showed bacterial growth and received targeted antibiotic therapy (Table 1). Preoperative ureteral stenting was performed in 25% (n = 10) of patients due to obstructive uropathy, while 75% (n = 30) proceeded directly to PCNL (Table 1).

Comorbidity profile

The majority of patients (75%, n = 30) had no significant comorbidities (Table 3). Among those with comorbidities, type 2 diabetes mellitus was most prevalent (12.5%, n = 5), followed by systemic hypertension (5%, n = 2). Body mass index was normal in 90% (n = 36) of patients (Table 1).

**Table 3:** Distribution of comorbid conditions among the study participants (n = 40)

Comorbidity	Number (n = 40)	Percentage (%)
None	30	75.0
Type 2 diabetes mellitus	5	12.5
Systemic hypertension	2	5.0
Chronic kidney disease	1	2.5
COPD	1	2.5
Coronary artery disease	1	2.5

## Discussion

This study provides evidence regarding the effects of PCNL on renal morphology and vascular resistance during a 12-18-month follow-up period, addressing an important gap in the current body of evidence. Our data demonstrate the preservation of renal morphology and arterial resistance following PCNL, supporting the procedure's standing as a safe, effective option for large renal stones.

Our findings align with previous research demonstrating PCNL safety. El-Nahas et al. [20] found no significant long-term deterioration in renal function post-PCNL, consistent with our observation of preserved serum creatinine levels (mean 1.14 ± 0.42 mg/dL). Akman et al. [21] utilized color Doppler ultrasonography and reported no significant functional changes but observed subtle morphological alterations. Our study extends these findings by demonstrating that 97.5% (n = 39) of patients maintained normal renal echogenicity, with only one patient showing hyperechogenic changes associated with pre-existing chronic kidney disease, compared to the contralateral kidney which showed normal echogenicity. This suggests morphological alterations are likely related to pre-existing conditions rather than PCNL itself.

The resistive index values observed in our study (operated kidney: 0.626 ± 0.042; contralateral kidney: 0.613 ± 0.040) fall well within normal physiological range, indicating preserved vascular perfusion with normal vascular resistance patterns. These findings suggest that properly performed PCNL does not significantly alter overall renal vascular resistance patterns. The maintained parenchymal thickness measurements (operated: 16.08 ± 1.05 mm; contralateral: 16.07 ± 0.96 mm) support morphological preservation following PCNL, addressing concerns about significant parenchymal loss from percutaneous access and stone manipulation. The statistical comparison revealed no significant differences between operated and contralateral kidneys (p > 0.05), indicating that PCNL does not cause measurable alterations in these parameters during the 12-18-month follow-up period.

Our demographic analysis revealed a predominance of middle-aged males (72.5%, n = 29), with the highest incidence in the 31-45-year age group (40%, n = 16), consistent with established epidemiological patterns of urolithiasis [1,22]. The male predominance likely reflects multifactorial influences including dietary habits, occupational factors, and genetic predisposition. The slight left-sided predominance (55%, n = 22) differs from some reports suggesting equal bilateral distribution, although clinical significance remains unclear [23].

The majority of patients (75%, n = 30) had no significant comorbidities, although the presence of diabetes mellitus (12.5%, n = 5) and hypertension (5%, n = 2) aligns with the literature identifying metabolic syndrome components as risk factors [24]. The 17.5% (n = 7) rate of positive preoperative urine cultures required appropriate antibiotic therapy to prevent postoperative complications. Preoperative stenting was performed in 25% (n = 10) of patients presenting with obstructive uropathy, allowing for infection control and renal function optimization [25,26].

Grey scale and color Doppler ultrasonography proved effective for assessing renal morphology and vascular parameters, with its accessibility, cost-effectiveness, and lack of radiation exposure making it ideal for follow-up studies [11,12]. The overall study results demonstrated favorable outcomes following PCNL procedures. With 97.5% (n = 39) of patients maintaining normal renal echogenicity and resistive index values within physiological ranges, the data supports the safety and efficacy of PCNL for large renal stone management. The single patient with abnormal findings had pre-existing chronic kidney disease, emphasizing the importance of preoperative renal assessment in patient selection and counseling. The preservation of renal function, as evidenced by stable serum creatinine levels and maintained vascular parameters, validates the minimally invasive nature of PCNL and its suitability for patients requiring intervention for large renal stones [27].

Our study offers reassuring evidence that PCNL does not compromise renal function during a 12-18-month follow-up period. The procedure causes minimal alterations in renal structure and vascular resistance, supporting its retention as the preferred treatment option for appropriately selected patients with large renal stones [4,28]. Nonetheless, longer follow-up, paired pre- and postoperative analysis, and the use of advanced imaging still merit examination before drawing definitive conclusions about long-term safety.

Limitations

Several important limitations should be acknowledged. First, resistive index measurements were obtained from interlobar and arcuate arteries in the mid-kidney region but did not specifically assess vascular changes at the exact puncture sites where the nephrostomy tract was created. PCNL typically involves puncturing one or two calyces, with potential injury to arcuate arteries. Localized vascular injury at these specific sites may occur despite preserved overall renal perfusion through compensatory mechanisms mediated by the renin-angiotensin-aldosterone system (RAAS). Future studies should employ targeted Doppler assessment of vessels at puncture sites to evaluate focal vascular injury.

Second, although experienced radiologists performed all imaging studies, no radiologist was included as a contributing author. This represents a limitation in the multidisciplinary collaboration, and future studies should ensure radiologist co-authorship for comprehensive expertise.

Third, the cross-sectional design limited our ability to compare pre- and postoperative parameters within individual patients. A prospective longitudinal design with baseline preoperative imaging would provide stronger evidence regarding PCNL's effects on renal morphology and vascular resistance. The modest sample size and single-center setting limit generalizability and may introduce selection bias.

Fourth, patients with solitary kidneys or those undergoing bilateral procedures were deliberately excluded, restricting the findings' applicability to these groups. Complete data regarding stone disease duration prior to PCNL were not available for all patients, limiting analysis of how chronic obstruction may influence post-operative findings.

Finally, standard ultrasound may overlook subtle morphologic changes that more sensitive modalities like MRI or contrast-enhanced CT would reveal. The 12-18-month follow-up period, while providing intermediate-term data, does not constitute true long-term assessment, and longer follow-up periods are warranted to fully evaluate the durability of these findings.

## Conclusions

This cross-sectional study shows that PCNL has minimal long-term effects on renal morphology and arterial vascular resistance 12-18-month post-op. Key evidence includes normal resistive index values (operated kidney: 0.624 ± 0.04; contralateral: 0.611 ± 0.037), preserved renal echogenicity in 97.5% of patients, and stable serum creatinine (1.14 ± 0.42 mg/dL).

These findings confirm PCNL as a safe, effective treatment for large renal stones, with low risk of long-term impairment. Comparable metrics between kidneys indicate no major changes to renal structure or blood flow. Clinicians can reassure patients and recommend PCNL as first-line therapy without concerns for lasting complications. Success depends on proper patient selection, skilled technique, and follow-up, including pre-op kidney evaluation, infection treatment, and staging if needed. Future studies should include longer follow-ups, prospective comparisons, advanced imaging, and subgroups like solitary kidneys or complex cases. Overall, this research strengthens PCNL's role in managing rising urolithiasis rates, providing effective stone removal with few long-term renal issues.
